# Eating and Drinking

**Published:** 1872-05

**Authors:** 


*Eating and Drinking*; a popular manual of food and diet in health and Disease. By GEORGE M. BEARD, M. D. New York: G. P. Putnam & Sons, 1871.

